# Supplementary material for: Genome-Wide Identification and Characterization of the Msr Gene Family in Alfalfa under Abiotic Stress
Source: Int J Mol Sci. 2023 Jun 1;24(11):9638. doi: 10.3390/ijms24119638 (PMC10253413; doi:10.3390/ijms24119638)
Supplement: Supplementary file 1 [file ijms-2370858-Supplementary Figure S1.pdf › ijms-2370858-Supplementary Figure S1.pdf]

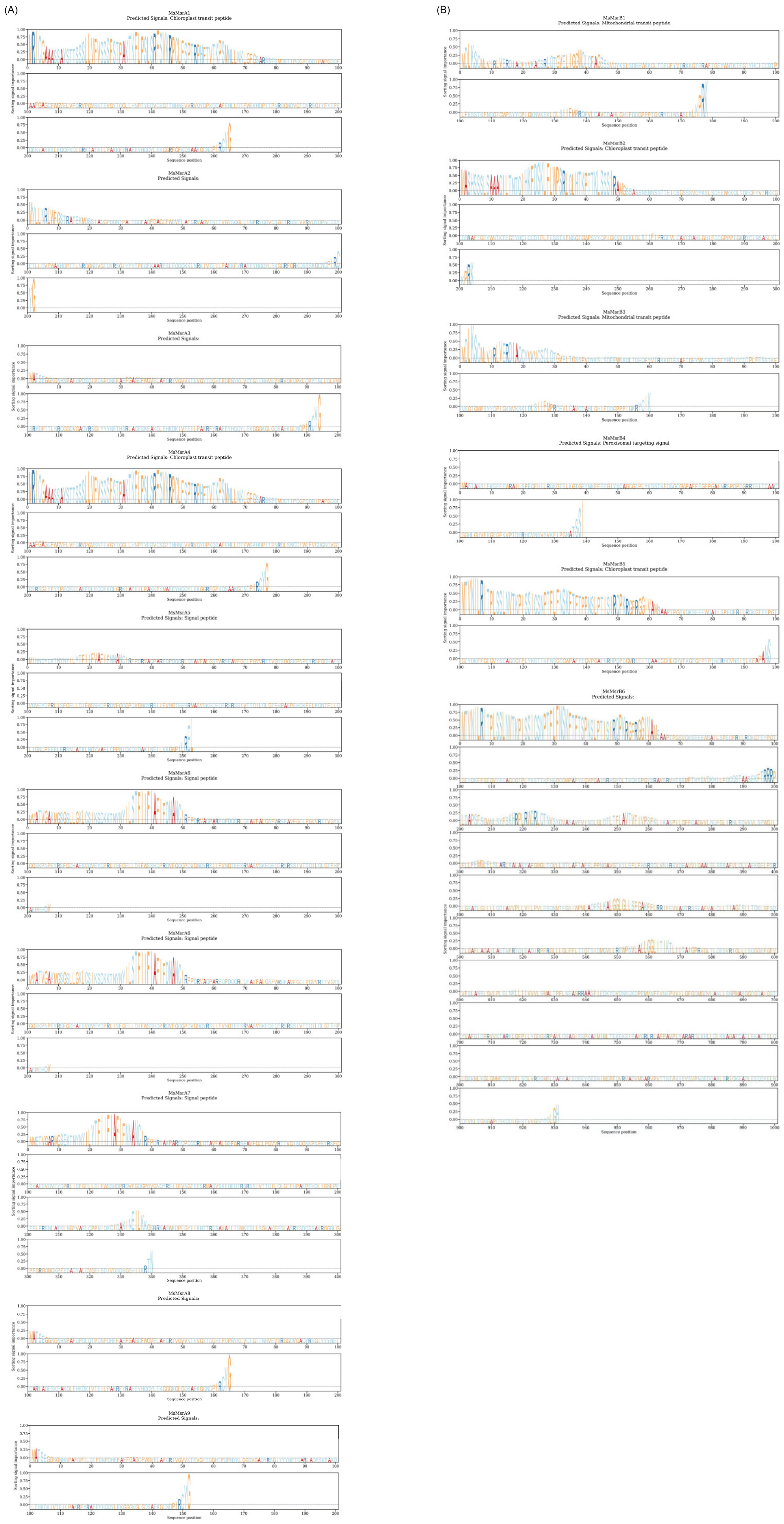

Figure S1. The predicted signal peptides for 15 MsMsr proteins are as follows: (**A**) 9 predicted signal peptides for MsMsrA proteins; (**B**) 6 predicted signal peptides for MsMsrB proteins.
